# Dystrophin-Deficient Muscular Dystrophy in a Family of Shiba Inu Dogs with a Complex Deletion Encompassing *DMD* Exon 5

**DOI:** 10.3390/genes16111369

**Published:** 2025-11-11

**Authors:** Laura Mcleay, Simone Hardinge, Katie M. Minor, Steven G. Friedenberg, Jonah N. Cullen, Ling T. Guo, G. Diane Shelton

**Affiliations:** 1 Small Animal Specialist Hospital, North Ryde 2113, Australia; lmcleay@sashvets.com; 2 Melbourne Veterinary Specialist Centre, Glen Waverley, Victoria 3250, Australia; shardinge@melbvet.com.au; 3 Department of Veterinary Clinical Sciences, College of Veterinary Medicine, University of Minnesota, St. Paul, MN 55108, USA; minork@umn.edu (K.M.M.); fried255@umn.edu (S.G.F.); cull0084@umn.edu (J.N.C.); 4 Comparative Neuromuscular Laboratory, Department of Pathology, University of California San Diego, La Jolla, CA 92093, USA; liguo@health.ucsd.edu

**Keywords:** canine, muscle, myopathy, acute death, whole genome sequencing, genetic disease

## Abstract

Background: Two Shiba Inu littermates presented for investigation of marked and persistent elevation of creatine kinase activities. Method and Results: Histopathology of muscle biopsy samples revealed a dystrophic phenotype and immunostaining confirmed an absence of dystrophin protein in both cases. Whole genome sequencing of one affected dog revealed a complex deletion in the *DMD* gene encompassing exon 5. Screening of 27 related dogs confirmed an X-linked inheritance. The variant was identified in three related male dogs. One littermate died from cardiac arrest and the other littermate had no clinical myopathic signs at the time of the manuscript’s preparation. An additional related male dog reportedly died suddenly during grooming. Conclusion: This study adds a new breed to the canine dystrophinopathy spectrum having a ~17 kb deletion that encompasses exon 5 of *DMD.* This same exon 5 deletion has been identified in human dystrophin-deficient muscular dystrophy patients.

## 1. Introduction

Muscular dystrophy encompasses a heterogeneous group of inherited myopathies characterized by progressive degeneration of striated muscle. These disorders are a result of variants in genes encoding structural or regulatory proteins critical to the integrity and function of the sarcolemma and associated cytoskeletal components [1]. Dystrophin-deficient muscular dystrophy (dystrophinopathy) is a major subset of muscular dystrophies, resulting from variants in the *DMD* gene, which encodes the dystrophin protein and is located on the short arm of the X chromosome [2]. Dystrophin is an essential cytoskeletal protein that links the actin cytoskeleton of muscle fibers to the extracellular matrix via the dystrophin–glycoprotein complex. Loss or functional deficiency of dystrophin results in sarcolemmal instability, increased susceptibility to mechanical stress, and subsequent myofiber necrosis. Clinically, dystrophin-deficient animals exhibit early onset, progressive muscular weakness, exercise intolerance, abnormal gait, dysphagia, macroglossia, respiratory weakness, and variable cardiomyopathy [3,4,5,6,7]. The prognosis is considered grave, with most affected dogs experiencing fatal outcomes before two years of age. The most common cause of death is cardiac or respiratory failure.

In humans, variants in *DMD* cause X-linked Duchenne muscular dystrophy (DMD) and Becker muscular dystrophy (BMD). DMD is the most common form of muscular dystrophy in people, with a global birth prevalence of 19.8 in 100,000 males [8]. BMD is a milder form of muscular dystrophy associated with reduced but not absent dystrophin and has a later onset and slower progression [1]. Thousands of *DMD* variants have been identified in people and approximately one third of children affected with DMD or BMD are a result of de novo germline mutations [2]. In veterinary medicine, dystrophin-deficient X-linked MD is best described in the golden retriever, which is caused by a splice site variant in the *DMD* gene and closely mimics the human Duchenne phenotype [6]. However, to date, at least 20 unique *DMD* variants have been identified across 15 different dog breeds, including point mutations and various chromosomal rearrangements, illustrating the genetic heterogeneity of dystrophinopathies [9].

Despite the expanding catalog of known *DMD* variants in dogs, dystrophinopathy has not been documented in the Shiba Inu breed. A single case report from 2018 described a Shiba Inu with suspected myotonic muscular dystrophy; however, the histological samples from this case were not subject to immunostaining for dystrophin or gene sequencing [10]. In the present study, we describe dystrophinopathy and identify the likely causative gene variant and inheritance pattern in a family of Shiba Inu dogs.

## 2. Materials and Methods

All clinical and diagnostic procedures performed on the animals in this study were with informed consent from the owners and performed by licensed veterinary surgeons. All DNA samples on related dogs were collected by buccal mucosal swab performed by the owner and with the owner’s consent. Where blood or muscle tissue was used for genetic analysis, the samples were archived from those already collected for diagnostic purposes under owner consent. Additional information about dogs related to the index cases was obtained from the breeder or referring veterinarian with owner consent.

### 2.1. Animals

The index cases (Case 1 and Case 2) are sibling Shiba Inu dogs who presented independently to separate private specialist veterinary hospitals for the investigation of incidental muscle enzyme elevation. Both dogs underwent diagnostic tests at the request of their owners. In addition, 4 related dogs with elevated creatine kinase (CK) activities (Cases 3–6) were clinically evaluated. Buccal mucosal swabs were collected from 27 related Shiba Inu dogs. Associated pedigree information was obtained from the breeder.

### 2.2. Clinical Examinations

Physical and neurological examinations were performed, and blood was collected for hematology, biochemistry, and electrolyte analysis. Point-of-care thoracic ultrasound and thoracic radiographs were performed on Case 1. Electromyography under general anesthesia was performed on Case 1 and included an assessment of masticatory, epaxial, pelvic, and thoracic appendicular muscles (including flexors and extensors at various depths) on the left side using digital electrodiagnostic equipment (Nicolet Viking Quest, Natus, Newington, NSW, Australia).

### 2.3. Histopathology and Immunostaining

Muscle samples were collected by an open biopsy procedure from the triceps brachii and biceps femoris muscles of Case 1 and cranial tibial and biceps femoris muscles of Case 2 under general inhalational anesthesia. Additional muscle samples were collected from the diaphragm, cardiac ventricular walls, and intercostal muscle of Case 1 post-mortem. Unfixed chilled and formalin-fixed muscle samples were submitted to the Comparative Neuromuscular Laboratory, University of California San Diego by a courier service. The unfixed muscle samples were evaluated in frozen sections using a standard panel of histochemical stains and reactions [11] and the fixed samples were evaluated in routine paraffin sections.

To characterize a specific form of muscular dystrophy, cryosections from the biceps femoris muscle of both cases and an archived control muscle were cut (8 μm) and stained for indirect immunofluorescence, as previously described [12]. Several monoclonal or polyclonal antibodies were used, including those against the rod (1:100, NCL-DYS1) and carboxy-terminus (1:100, NCL-DYS2) of the dystrophin, and against the utrophin (1:20, NCL-DRP2), developmental myosin heavy chain (dMHC1:20, NCL-dMHC), α-sarcoglycan (1:200, gift of Eva Engvall) [13], β-dystroglycan (1:100, NCL, bDG), laminin α2 (gift of Eva Engvall, 4F11, direct apply) [14], and collagen VI (gift of Eva Engvall, 3G7, direct apply) [15]. All NCL primary antibodies are from Leica Biosystems, Deer Park, IL, USA. 

### 2.4. Genetic Investigations

Genomic DNA was prepared from frozen muscle archived on Case 1 and Case 2 using the Qiagen DNEasy kit (Qiagen, Germantown, MD, USA) according to the package’s instructions. A DNA library for whole genome sequencing (WGS) from Case 1 was prepared using an Illumina TruSeq PCR-Free kit (Ilumina, Inc., San Diego, CA, USA) and 150 base-pair, paired-end reads were generated on an Illumina 6000 sequencer by Azenta Life Sciences (South Plainfield, NJ, USA). A total of 752.1 million paired-end reads were generated, corresponding to a mean 44X genome-wide coverage. Sequence reads were mapped against the dog reference genome UU Cfam GSD 1.0 [16,17] and processed using the OnlyWAG pipeline, as described [18]. Raw sequence reads are available in the NCBI Short Read Archive under accession number SRR34787340 (BioProject PRJNA937381).

WGS variants from the affected dogs were compared to an internal WGS database developed at the University of Minnesota containing 3023 dogs, wolves, and coyotes of 402 diverse breeds; this database includes 1971 dogs, wolves, and coyotes released by the Dog10K consortium [17]. The WGS data from these 3023 dogs was processed using the same bioinformatics pipeline referenced above. Variants unique to the affected dogs were prioritized by the predicted consequence and impacted by a Variant Effect Predictor [19]. High (e.g., frame shift, loss or gain of stop or start codon, affecting a splice junction) and moderate impact (e.g., missense) variants were evaluated for further investigation. A list of these unique variants is provided in Supplementary Table S1.

### 2.5. Long-Read PCR Sequencing

Borders of the deleted *DMD* sequence and characterization of the insertion were determined by long-read NGS-based PCR sequencing utilizing Oxford Nanopore Technologies by Azenta Life Sciences (South Plainfield, NJ, USA). A Takara PrimeSTAR GXL DNA polymerase (Takara Bio, San Jose, CA, USA) PCR reaction was prepared according to the manufacturer’s standard protocol with forward primer 5′-CCCCACTGAGAAACCACACT and reverse primer 5′-CCAAGTCCACAAGAGCCAAT at an annealing temperature of 60 °C with a 3 min extension time, which produced a 2689 bp amplicon for Case 2.

Genotyping of other dogs was performed via agarose gel electrophoresis visualization for the presence/absence of two amplicons: one containing DMD exon 5 utilizing forward primer 5′-TTACCTGCCAGTGGAGGATTAT and reverse primer 5′-AAATGCAGTCACCCCTAATTGT to produce a 516 bp product, and one spanning the ~17 kb deletion utilizing forward primer 5′-ATGGTGGGTATTTGTCGTTTC and reverse primer 5′-TATGGTGATTCAAGGACACAGG to produce a 905 bp product. Both reactions were prepared with Qiagen HotStarTaq Polymerase (Qiagen, Germantown, MD, USA) according to the manufacturer’s recommended reaction protocol with an annealing temperature of 64 °C and an extension time of 30 s.

## 3. Results

### 3.1. Animals/Clinical Evaluation

**Figure 1:**
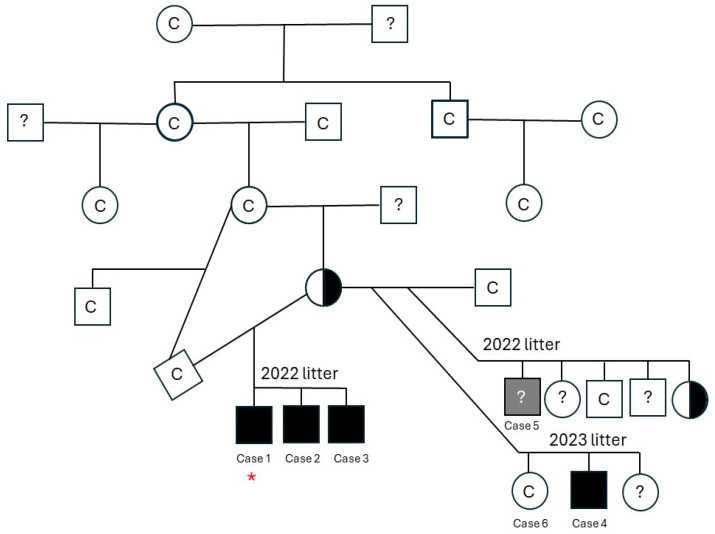
Pedigree information of the affected family, where available, is displayed. Whole genome sequencing was performed on Case 1 which is noted with a red star. Dogs with a “C” are clear for the deletion, dogs that were not genotyped are denoted with a “?”, affected males are filled squares, and carrier females are half-filled circles. The gray square is the presumed case (Case 5) that died prior to DNA collection, thus the genotype unknown.

Pedigree information of the affected family, where available, is displayed in Figure 1. The WGS for Case 1 is noted with a red star, dogs with a “C” are clear for the deletion, dogs that were not genotyped are denoted with a “?”, affected males are filled squares, and carrier females are half filled. Clinical, diagnostic, treatment, and outcome information was available for Cases 1–6.

#### 3.1.1. Case 1

A 16-month-old male Shiba Inu dog was referred for investigation of markedly elevated alanine aminotransferase (ALT) activity (742 U/L; reference interval [RI]: 10–125 U/L) identified incidentally during a routine pre-anesthetic blood evaluation prior to elective castration. At that time, the dog was clinically normal with a normal physical examination and a body condition score (BCS) of 5/9. Other liver-associated biochemical parameters were within reference ranges. A comprehensive hepatic evaluation, including pre- and post-prandial bile acids, abdominal ultrasonography, urinalysis, and urine culture was unremarkable. Treatment was initiated with a hepatoprotective supplement, HepatoAdvanced^®^ (containing S-adenosylmethionine, silybin, and N-acetylcysteine), and a follow-up evaluation was recommended after 4 weeks.

At the 4-week follow-up serum biochemistry revealed a persistent elevation of ALT (750 U/L), marked elevation of CK (95,726 U/L; RI: 73–510 U/L), and aspartate aminotransferase (AST; 2158 U/L; RI: 18–80 U/L) activities. Hyperkalemia was also documented (serum potassium 10.2 mmol/L; RI: 3.9–5.9 mmol/L). The dog was referred to an internal medicine service for further investigation.

At referral, the dog remained alert and responsive but demonstrated mild increased respiratory effort at rest. Point-of-care thoracic ultrasonography revealed no pleural or pulmonary abnormalities. Repeat bloodwork showed normalization of potassium (3.5 mmol/L). Hematologic abnormalities included mild, normocytic anemia (Hb 119 g/L; RI: 131–205 g/L) and discrepancies in mean corpuscular volume (MCV 73.7 fL; RI: 61.6–73.5) and mean corpuscular hemoglobin concentration (MCHC 260 g/L; RI: 320–379). On manual blood smear evaluation by a veterinary pathologist, blood cells appeared subjectively smaller than expected, consistent with breed-associated microcytosis. This finding contrasted with the marginally increased mean corpuscular volume (MCV) reported by the automated hematology analyzer, suggesting that the elevated MCV was likely artifactual.

At a further 4-week follow-up, the owner reported progressive lethargy, reduced exercise tolerance, and a 500 g weight loss. On physical examination, increased respiratory effort persisted with loss of body condition and mild generalized muscle atrophy. No neurological deficits were identified. Biochemistry revealed sustained elevations in CK (48,604 U/L), ALT (435 U/L), and AST (973 U/L) activities, and potassium was again elevated (6.9 mmol/L; RI: 4.3–5.9 mmol/L). Hematology showed persistent mild anemia. Resting cortisol was slightly elevated (135 nmol/L; RI: 30–100 nmol/L). Urinalysis identified hemoglobinuria. Thoracic radiographs and electrocardiographic evaluations were unremarkable.

Electromyographic (EMG) evaluation of Case 1 revealed spontaneous fibrillation potentials and prolonged insertional activity in all muscle groups tested, which included the appendicular muscles of the thoracic and pelvic limb, the epaxial, and masticatory muscles. Complex repetitive discharges were observed in the triceps brachii, biceps brachii, quadriceps muscle group, biceps femoris, and temporalis muscles. The EMG findings were consistent with a generalized myopathy. Samples were obtained from the right triceps brachii and biceps femoris muscles via an open biopsy procedure under general anesthesia. Cardiac arrest occurred shortly after recovery from general anesthesia and the dog could not be recovered. A limited post-mortem examination focused on the thorax. Multiple right-sided rib fractures were noted, attributed to chest compressions and diffuse pulmonary hemorrhage and edema were observed. There were no gross abnormalities of the heart or great vessels. No valvular, pericardial, or myocardial lesions were identified. Tissue samples from the left and right ventricular walls, ventricular septum, diaphragm, and intercostal muscles were collected and submitted for histopathology along with antemortem muscle samples.

#### 3.1.2. Case 2

A 16-month-old male entire Shiba Inu, a full sibling to Case 1, was referred to an internal medicine specialist for investigation of markedly elevated CK activity (45,868 U/L; RI 73–522 U/L), as well as elevated ALT (1214 U/L; RI 13–98 U/L) and AST (1035 U/L; RI: 17–84 U/L) activities, identified during an episode of self-limiting gastrointestinal signs. Hematology revealed a mild reticulocytosis (118 × 10^9^/L, RI 10–90 × 10^9^/L) and a mild lymphocytosis (5.1 × 10^9^/L; RI 0.9–4.1 × 10^9^/L). Biochemistry was repeated three weeks later, with ongoing elevations in CK (32,920 U/L), ALT (772 U/L), and AST (833 U/L) activity. The owner expressed intermittent concerns over the previous 11 months (since the age of 5 months) about the pelvic limb gait and perceived pain following activity. The dog was treated with fluoxetine 10 mg once daily for anxiety and gabapentin 100 mg twice daily for perceived discomfort and was on these medications at the time of referral.

At referral, the dog was bright and alert and was noted to have a mildly increased respiratory rate and effort at rest. Lameness was not obvious. Musculoskeletal examination identified a grade 2 left medial luxating patella with no other abnormalities. Neurological exam showed no deficits. The dog was noted to be hesitant moving up stairs and displayed an occasional ‘bunny-hopping’ gait when descending stairs.

Repeat biochemistry showed persistently and markedly elevated CK (49,999 U/L), ALT (897 UL) and AST (968 U/L) activities. A whole-body CT scan was performed. A thickened diaphragmatic margin and mild lymphadenopathy affecting the cranial mediastinal, medial iliac, sacral, and popliteal lymph nodes were reported. *Toxoplasma gondii* and *Neospora caninum* antibody titers were negative.

Muscle biopsies were obtained under general anesthesia from the left and right cranial tibial muscles and left and right biceps femoris muscles. Surgical castration was performed under the same anesthetic, which was considered routine. Recovery from these procedures was without complication.

At the time of this writing, approximately 1 year after initial referral, the dog continues to do well with occasional stiff gait and mild pelvic limb muscle atrophy. CK activity remained elevated at 21,223 IU/L.

#### 3.1.3. Case 3

A 16-month-old male full sibling of Cases 1 and 2 was evaluated for polyuria, polydipsia, and poor weight gain. Hematology revealed mild non-regenerative anemia (RBC 4.72 × 10^12^/L, RI: 5.65–8.87 × 10^12^/L, HCT 0.328 L/L, RI: 0.373–0.617). Biochemical testing showed elevated CK activity (142,951 U/L, RI: 73–510), ALT activity (500 U/L, RI: 8–75 U/L), AST activity (1939 U/L, RI: 18–80), and potassium (9.8 mmol/L, RI: 3.9–5.9). Hyposthenuria, hematuria, and pyuria were found on urinalysis, urine protein–creatinine ratio was normal (<0.2) and the urine culture was negative. No underlying cause for urinary abnormalities or polyuria and polydipsia was found and repeated urine specific gravity was normal on three subsequent measurements. The polyuria and polydipsia resolved; however, the dog developed intermittent pelvic limb paresis, stiff gait, and poor muscle mass. Repeat biochemical evaluation 16 months later revealed persistently elevated CK (35,243 U/L) and ALT (884 U/L) activities. The dog was chemically sterilized following the diagnosis of muscular dystrophy.

#### 3.1.4. Case 4

An approximately 2-year-old male castrated half sibling was presented for evaluation after possibly ingesting ibuprofen. Biochemical evaluation showed elevated ALT (990 U/L, RI: 10–125 U/L). Further investigation or repeat biochemical analysis was declined by the owner. The dog has since developed a stiff gait, increased respiratory effort, and weight loss.

#### 3.1.5. Case 5

A 1-year-old male half sibling was reported to have had sudden death during grooming. Gross evaluation of the heart did not reveal any abnormalities; however, histopathology was not performed. Tissues were not available for genetic testing.

#### 3.1.6. Case 6

An approximately 2-year-old female half sibling (immediate sibling to Case 4) was reported as clinically normal, but serum biochemistry showed mildly elevated CK activity (598 U/L, RI: 73–510 U/L). The dog was clinically normal, and no further investigations were performed. The dog remains clinically normal at the time of this writing.

### 3.2. Histopathology and Immunohistochemistry (Cases 1 and 2)

**Figure 2:**
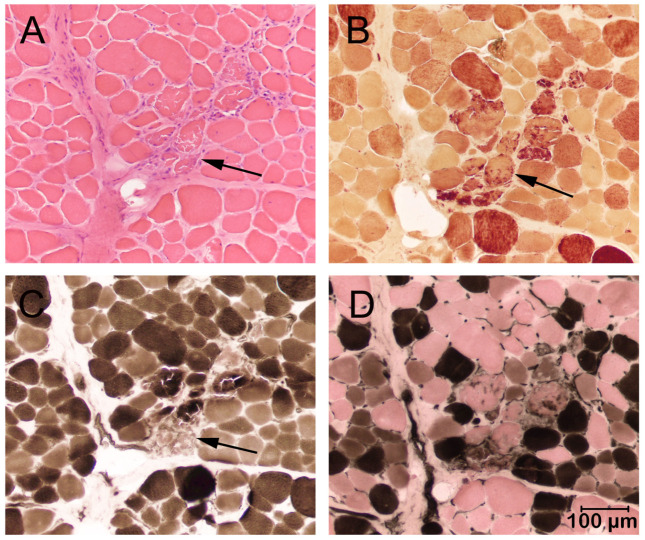
Cryosections (8 µm) from the biceps femoris muscle of Case 2. (**A**). Variability in myofiber size was present with multifocal areas of myonecrosis (arrow, H and E stain). (**B**). Necrotic fibers underwent phagocytosis (arrow, esterase reaction). (**C**,**D**). Groups of necrotic fibers (arrow, **C**) and regenerating type 2C fibers (light brown stained fibers in (**D**)) were highlighted with the ATPase reaction at pH 9.8 (**C**) and 4.3 (**D**). These changes are consistent with a dystrophic phenotype. Bar in lower right = 100 µm for all images.

In both cases, a marked variability in myofiber size was present in all skeletal muscles sampled with multifocal groups of necrotic degenerating fibers and clusters of regenerating type 2C fibers consistent with a dystrophic phenotype. Representative images from Case 1 are shown in Figure 2.

**Figure 3:**
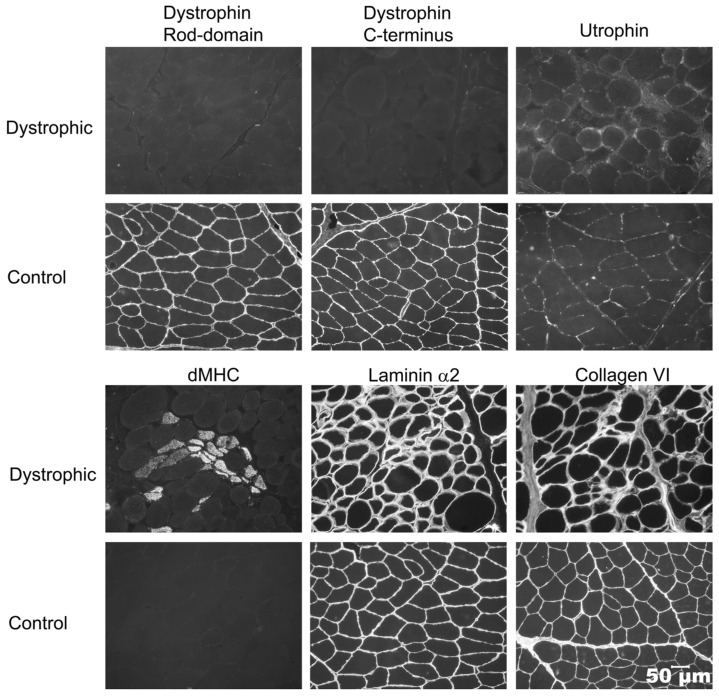
Immunofluorescent staining of cryosections from the quadriceps muscle of Case 1 and an archived control muscle using monoclonal antibodies against the rod and carboxy terminus of dystrophin, against utrophin and developmental myosin heavy chain (dMHC) for regenerating fibers, and against laminin α2 and collagen VI. Compared to control tissue, staining for both the rod domain and carboxy terminus of dystrophin was absent. Staining for utrophin increased and clusters of regenerating fibers were shown using the antibody against dMHC. Antibody staining for laminin α2 and collagen VI was similar to the control. Staining from Case 1 is shown with similar changes to Case 2. Bar in lower right = 50 µm for all images.

Immunofluorescent staining was performed on cryosections from Case 1, Case 2, and archived control muscle to determine a specific form of muscular dystrophy (Figure 3). Antibody staining of dystrophin rod-domain and C-terminus proteins were absent or markedly decreased compared to control muscle. Utrophin staining increased on the muscle’s sarcolemma. Staining for regenerating fibers using the monoclonal antibody against developmental myosin heavy chain highlighted regenerating clusters. Staining for both laminin α2 and collagen VI proteins was similar to the control. Based on these staining’s, a diagnosis of dystrophin-deficient muscular dystrophy was made in both cases

### 3.3. Genetic Testing

**Figure 4:**
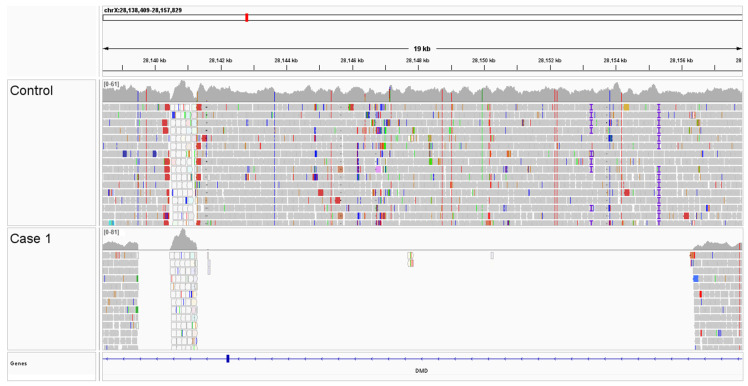
*DMD* deletion encompassing exon 5 detected with whole genome sequencing of Case 1 compared to a control genome. A 19 kb bp segment flanking the deletion on chromosome X is shown.

A comparison of WGS variants from Case 1 were compared to an internal WGS database containing 3023 dogs, wolves, and coyotes, with no unique protein-coding *DMD* variants identified. However, visual inspection of aligned reads identified an approximately 17 kb deletion that encompasses *DMD* exon 5, resulting in a presumptive in-frame deletion of 31 amino acids (NM_001003343.1, p.Val89_Gln119del) (Figure 4). Borders of the deleted *DMD* sequence (chrX:28,139,503_28,156,189del) and characterization of a partial LINE insertion were determined by long-read NGS-based PCR sequencing (Supplementary Table S2).

Genotyping of related dogs via the presence/absence visualization of PCR amplicons confirmed that all affected male cases with available DNA (Case 1–4) lacked the *DMD* exon 5 sequence. The cases’ dam and one female littermate were found to be carriers of the *DMD* exon 5 deletion; all other dogs were clear of the deletion. Interestingly, the maternal grandmother of the affected dogs was clear of the deletion, however the maternal grandsire was not available for genotyping.

## 4. Discussion

This study describes a novel deletion in *DMD* gene exon 5 that is likely causative of dystrophin-deficient muscular dystrophy in a family of Shiba Inu dogs with X-linked inheritance. Exon 5 is 93 base pairs long and results in an in-frame deletion of 31 amino acids. The same variant has been described in people with both mild and severe dystrophic phenotypes [20]. Of further interest, the carrier dam’s mother was free of the mutation. This finding suggests a spontaneous mutation in the *DMD* gene that happened by chance in the egg or very early in embryonic development in the carrier dam, given the propensity of the *DMD* gene to mutations [2]. Since the dam has one copy of the dystrophin gene with a mutation and one copy without a mutation, the dam is a carrier (Figure 1). The carrier dam passed a copy of the muted *DMD* gene to the affected male offspring. An alternative explanation is that the mutation arose in the maternal grandmother’s or grandfather’s germline cells; however, none of the siblings were available to test. A similar situation where the parents were clear of the variant was reported in a litter of American Staffordshire terriers with a mutation in the *EXT2* gene associated with osteochondromatosis. Multiple affected puppies had two clear parents [21].

Of clinical relevance is the striking difference in clinical presentations among the affected dogs ranging from progressive mild muscle atrophy, exercise intolerance, respiratory fatigue, and death due to cardiac arrest following routine anesthesia in Case 1 to unexplained persistently elevated CK activities in Case 2 without obvious neuromuscular deficits. A common finding in all affected dogs was markedly and persistently elevated CK activity. In both cases where muscle biopsy samples were collected, a degenerative and regenerative myopathy consistent with a dystrophic phenotype was found (Figure 2). A specific type of muscular dystrophy cannot be determined by the histological phenotype; thus, both cases were confirmed to have an absence of dystrophin protein on immunofluorescence staining using antibodies against dystrophy-associated proteins (Figure 3).

In general, the clinical signs at presentation were mild or not obvious in Cases 1–3 in comparison to well-documented golden retriever muscular dystrophy [6] and DMD in people [1]. A mild form of dystrophinopathy has been identified in Labrador retrievers with persistently elevated CK activity first noted at the time of neuter without clinically evident weakness or stiffness [22]. The long-term outcome was determined in this study for 13 mildly affected male Labrador retrievers: four dogs died acutely of cardiomyopathy and arrhythmias at 4–5 years of age, two dogs were euthanized for disseminated neoplasia at 6 and 7 years of age, and the remaining seven dogs were either euthanized for age-related diseases or were still alive at 11–12 years of age. The variable severity of clinical phenotypes in canine models and human dystrophin-deficient muscular dystrophy is well known [23], which can be a complication in the interpretation of clinical trials. Case 2 remains clinically normal at the time of this report, approximately 1 year post diagnosis.

Case 3 underwent investigations into polyuria and polydipsia, with no cause identified. To our knowledge, there is no clinical association between polyuria or polydipsia with muscular dystrophy. This case has since developed mild myopathic signs at 2.5 years of age, with no significant impact on daily activity or quality of life. The clinical presentation of Case 4 is like Case 1, with reported weight loss and increased respiratory effort. Unfortunately, Case 4 has limited diagnostic information with no available CK activity; however, the clinical signs are presumed to be caused by muscular dystrophy as this dog was also positive for the *DMD* variant. Case 6 was not a carrier of the *DMD* variant despite having CK elevation detected at screening. This degree of CK elevation is mild and may be attributable to venipuncture. This dog remains clinically normal at the time of this writing.

The *DMD* gene is the largest in the human genome, comprising 1.5% of the entire X chromosome, making it more susceptible to spontaneous mutations [2]. Most cases of DMD in people are inherited from the mother; however, spontaneous *DMD* mutations occur in approximately a third of cases. As the *DMD* gene is located on the X chromosome, the phenotype is expressed predominantly in males, with a 50% chance of inheritance from a carrier female. In cases where there is a high proportion of X-inactivation of the unaffected X chromosome, females can also be clinically affected with dystrophinopathy, although typically with a milder phenotype. In the majority of cases, females are asymptomatic carriers [24]. Genotyping and pedigree analysis in this cohort of dogs showed the dam was a carrier and one other related female was also a carrier. With the diagnosis and genetic basis of this dystrophinopathy established, all affected and carrier animals were sterilized, effectively halting further transmission of this condition.

A reported human exon 5 in-frame deletion mutation supports our conclusions that our exon 5 deletion is likely causative [20]. Various known independent exon 5 deletion mutations result in a variety of phenotypes from mild to severe. Immunofluorescent staining shown in Figure 1 [20] from a human patient with an exon 5 deletion using the same DYS1 and DYS2 antibodies used in this paper showed decreased protein staining for DYS1, barely detectable protein staining for DYS2, and undetectable protein staining for DYS3, so amounts of dystrophin protein can be variable from decreased to undetectable. It cannot be said with certainty that there must be detectable expression based on “predicted” or “expected” splicing around exon 5, and it cannot be said for certain what the cell will do with an mRNA missing exon 5 or with a protein missing a portion of the sequence. In either or both cases, destruction of the mRNA or protein would nicely explain the lack of protein detection. An RNAseq dataset would help address whether splicing defects result, but this is beyond the aim of this manuscript.

Case 1 in this study died suddenly following recovery from anesthesia, which was presumably due to cardiac arrest. Histopathologic examination of cardiac muscle samples collected post-mortem did not identify any abnormalities. Case 5 in this study died suddenly during a routine nail clip at a veterinary clinic at 1 year of age. Post-mortem evaluation performed by the veterinary surgeon identified no gross abnormalities; however, histopathologic evaluation was not performed. The cause of cardiac arrest in Case 5 was not determined and tissue was not available for genotyping. However, in light of the *DMD* variant identified in three related half siblings and one full sibling, dystrophinopathy is highly likely. Unfortunately, CK activity was not available.

Cardiomyopathy is a well-recognized component of DMD in people and since the application of mechanical ventilatory support, heart failure, or sudden cardiac arrest is now the leading cause of death in these patients [25,26]. Importantly, there are parallels between progressive cardiomyopathy in dystrophic dogs and boys with DMD [27]. Myocardial degeneration, regeneration, and fibrosis lead to progressive dilated cardiomyopathy with decreased ventricular ejection fractions and tachyarrhythmias secondary to myocardial fibrosis. Case 1 of this study showed no clear evidence of cardiac disease on physical examination, ECG, thoracic radiographs, or cardiac muscle sampling. For future cases where the CK activity is persistently elevated and a myopathy suspected, screening of cardiac function is recommended, including pre-anesthetic echocardiography, Holter monitoring, and measurement of serum troponin-I concentration. Serum troponin-1 concentration can be used as a biomarker for the early detection of cardiomyopathy in dystrophinopathy [28]. Prolonged post-anesthetic ECG monitoring should also be considered for myopathic patients.

The increased blood potassium in Cases 1 and 3 had been attributed to breed-related pseudohyperkalemia. A significant proportion of Shiba Inu dogs have been shown to have a high concentration of intracellular potassium in erythrocytes as well as erythrocyte osmotic fragility [29]. Leakage of intracellular potassium following blood collection can be misinterpreted as true hyperkalemia. A high concentration of intracellular potassium can cause the osmotic diffusion of isotonic diluent into red blood cells during automated analysis, causing in vitro cell swelling and an overestimation of MCV, so the high MCV in Case 1 is further supportive of pseudohyperkalemia. In chronic muscle disorders like muscular dystrophy, ongoing potassium leakage from myofiber degeneration is normally compensated by renal elimination. Hyperkalemia is not a feature of DMD or BMD in people, unless there is an inciting cause that exacerbates sarcolemmal instability such as volatile inhalant anesthetic agents or neuromuscular blocking agents [30]. Hyperkalemia is documented in human patients with muscular dystrophy under inhalant anesthesia and occurs secondary to rhabdomyolysis [30]. Inhalant anesthetic agents trigger increased intracellular calcium release from the sarcoplasmic reticulum and can exacerbate membrane instability in dystrophin-deficient muscle fibers, leading to rhabdomyolysis, potassium release, and hyperkalemic cardiac arrest [30]. Total intravenous anesthesia is recommended in people with dystrophin-deficient muscular dystrophy and should also be considered for dogs undergoing general anesthesia if a myopathy is suspected.

## 5. Conclusions

This study identifies a novel *DMD* variant in Shiba Inu dogs that is likely causative for X-linked muscular dystrophy in this breed. Marked and persistent elevations of CK activity should alert the clinician to the possibility of a muscular dystrophy with or without classical clinical signs. Given the association of cardiomyopathy with this form of dystrophy, cardiac screening is suggested. Careful perioperative monitoring of animals with X-linked muscular dystrophy is warranted. Genetic testing of dogs of susceptible breeds should be performed prior to breeding and carriers should be eliminated.

## Data Availability

Raw sequence reads are available in the NCBI Short Read Archive under accession SRR34787340 (BioProject PRJNA937381).

## Supplementary Materials

The following supporting information can be downloaded at: https://www.mdpi.com/article/10.3390/genes16111369/s1, Table S1: List of unique coding variants for Case 1; Table S2: Deleted sequence from Case 1 in red text.

## Abbreviations

The following abbreviations are used in this manuscript:

ALT	Alanine aminotransferase
AST	Aspartate aminotransferase
BMD	Becker muscular dystrophy
BCS	Body condition score
CK	Creatine kinase
dMHC	Developmental myosin heavy chain
DMD	Dystrophin deficient muscular dystrophy
EMG	Electromyogram
MCHC	Mean corpuscular hemoglobin concentration
MCV	Mean corpuscular volume
MD	Muscular dystrophy
NGS	Next generation sequencing
WGS	Whole genome sequencing

## References

[1] Hoang T., Dowdy R.A.E. (2024). A Review of Muscular Dystrophies. Anesth. Prog..

[2] Muntoni F., Torelli S., Ferlini A. (2003). Dystrophin and mutations: One gene, several proteins, multiple phenotypes. Lancet Neurol..

[3] Beltran E., Shelton G.D., Guo L.T., Dennis R., Sanchez-Masian D., Robinson D., De Risio L. (2015). Dystrophin-deficient muscular dystrophy in a Norfolk terrier. J. Small Anim. Pract..

[4] Sakai K., Motegi T., Chambers J.K., Uchida K., Nishida H., Shimamura S., Tani H., Shimada T., Furuya M. (2022). Dystrophin-deficient muscular dystrophy in a Toy Poodle with a single base pair insertion in exon 45 of the Duchenne muscular dystrophy gene. J. Vet. Med. Sci..

[5] Stevens R., Kanazono S., Petesch S., Guo L.T., Shelton G.D. (2022). Dystrophin-Deficient Muscular Dystrophy in Two Male Juvenile Brittanys. J. Am. Anim. Hosp. Assoc..

[6] Kornegay J.N. (2017). The golden retriever model of Duchenne muscular dystrophy. Skelet. Muscle.

[7] Barthélémy I., Calmels N., Weiss R.B., Tiret L., Vulin A., Wein N., Peccate C., Drougard C., Beroud C., Deburgrave N. (2020). X-linked muscular dystrophy in a Labrador Retriever strain: Phenotypic and molecular characterisation. Skelet. Muscle.

[8] Crisafulli S., Sultana J., Fontana A., Salvo F., Messina S., Trifirò G. (2020). Global epidemiology of Duchenne muscular dystrophy: An updated systematic review and meta-analysis. Orphanet J. Rare Dis..

[9] Shelton G.D., Minor K.M., Friedenberg S.G., Cullen J.N., Guo L.T., Mickelson J.R. (2023). Current Classification of Canine Muscular Dystrophies and Identification of New Variants. Genes.

[10] Shiga T., Okuno S., Uchida K., Chambers J.K., Nakayama H. (2018). Electrophysiological and histopathological findings of muscular disease suspected as myotonic dystrophy in a Shiba dog. J. Vet. Med. Sci..

[11] Dubowitz V., Sewry C.A., Oldfors A. (2021). Histological and histochemical stains and reactions. Muscle Biopsy: A Practical Approach.

[12] Guo L.T., Moore S.A., Forcales S., Engvall E., Shelton G.D. (2010). Evaluation of commercial dysferlin antibodies on canine, mouse and human skeletal muscle. Neuromuscul. Disord..

[13] Liu L.A., Engvall E. (1999). Sarcoglycan isoforms in skeletal muscle. J. Biol. Chem..

[14] Leivo I., Engvall E. (1988). Merosin, a protein specific for basement membranes of Schwann cells, striated muscle, and trophoblast, is expressed late in nerve and muscle development. Proc. Natl. Acad. Sci. USA.

[15] Hessle H., Engvall E. (1984). Type VI collagen. Studies on its localization, structure and biosynthetic form with monoclonal antibodies. J. Biol. Chem..

[16] Wang C., Wallerman O., Arendt M.-L., Sundström E., Karlsson Å., Nordin J., Mäkeläinen S., Pielberg G.R., Hanson J., Ohlsson Å. (2021). A novel canine reference genome resolves genomic architecture and uncovers transcript complexity. Commun. Biol..

[17] Meadows J.R., Kidd J.M., Wang G.D., Parker H.G., Schall P.Z., Bianchi M., Christmas M.J., Bougiouri K., Buckley R.M., Hitte C. (2023). Genome sequencing of 2000 canids by the Dog10K consortium advances the understanding of demography, genome function and architecture. Genome Biol..

[18] Cullen J.N., Friedenberg S.G. (2023). Whole animal genome sequencing: User-friendly, rapid, containerized pipelines for processing, variant discovery, and annotation of short-read whole genome sequencing data. G3.

[19] McLaren W., Gil L., Hunt S.E., Riat H.S., Ritchie G.R.S., Thormann A., Flicek P., Cunningham F. (2016). The Ensembl Variant Effect Predictor. Genome Biol..

[20] Toh Z.Y., Thandar Aung-Htut M., Pinniger G., Adams A.M., Krishnaswarmy S., Wong B.L., Fletcher S., Wilton S.D. (2016). Deletion of Dystrophin In-Frame Exon 5 Leads to a Severe Phenotype: Guidance for Exon Skipping Strategies. PLoS ONE.

[21] Friedenberg S.G., Vansteenkiste D., Yost O., Treeful A.E., Meurs K.M., Tokarz D.A., Olby N.J. (2018). A de novo mutation in the EXT2 gene associated with osteochondromatosis in a litter of American Staffordshire Terriers. J. Vet. Intern. Med..

[22] Shelton G.D., Minor K.M., Vieira N.M., Kunkel L.M., Friedenberg S.G., Cullen J.N., Guo L.T., Zatz M., Mickelson J.R. (2022). Tandem duplication within the DMD gene in Labrador retrievers with a mild clinical phenotype. Neuromuscul. Disord..

[23] Kornegay J.N., Spurney C.F., Nghiem P.P., Brinkmeyer-Langford C.L., Hoffman E.P., Nagaraju K. (2014). Pharmacologic management of Duchenne muscular dystrophy: Target identification and preclinical trials. ILAR J..

[24] Giliberto F., Radic C.P., Luce L., Ferreiro V., de Brasi C., Szijan I. (2014). Symptomatic female carriers of Duchenne muscular dystrophy (DMD): Genetic and clinical characterization. J. Neurol. Sci..

[25] Lechner A., Herzig J.J., Kientsch J.G., Kohler M., Bloch K.E., Ulrich S., Schwarz E.I. (2023). Cardiomyopathy as cause of death in Duchenne muscular dystrophy: A longitudinal observational study. ERJ Open Res..

[26] D’Amario D., Arcudi A., Narducci M.L., Novelli V., Canonico F., Parodi A., Dell’eRa G., Di Francesco M., Laborante R., Borovac J.A. (2025). Arrhythmic Risk Stratification and Sudden Cardiac Death Prevention in Duchenne Muscular Dystrophy: A Critical Appraisal. Rev. Cardiovasc. Med..

[27] Guo L.J., Soslow J.H., Bettis A.K., Nghiem P.P., Cummings K.J., Lenox M.W., Miller M.W., Kornegay J.N., Spurney C.F. (2019). Natural History of Cardiomyopathy in Adult Dogs with Golden Retriever Muscular Dystrophy. J. Am. Heart Assoc..

[28] Yamaguchi H., Awano H., Yamamoto T., Nambu Y., Iijima K. (2022). Serum cardiac troponin I is a candidate biomarker for cardiomyopathy in Duchenne and Becker muscular dystrophies. Muscle Nerve.

[29] Maede Y., Amano Y., Nishida A., Murase T., Sasaki A., Inaba M. (1991). Hereditary high-potassium erythrocytes with high Na, K-ATPase activity in Japanese shiba dogs. Res. Vet. Sci..

[30] Segura L.G., Lorenz J.D., Weingarten T.N., Scavonetto F., Bojanić K., Selcen D., Sprung J. (2013). Anesthesia and Duchenne or Becker muscular dystrophy: Review of 117 anesthetic exposures. Paediatr. Anesth..

