# Supplementary material for: Dystrophin-Deficient Muscular Dystrophy in a Family of Shiba Inu Dogs with a Complex Deletion Encompassing DMD Exon 5
Source: Genes (Basel). 2025 Nov 11;16(11):1369. doi: 10.3390/genes16111369 (PMC12651955; doi:10.3390/genes16111369)
Supplement: Supplementary file 1 [file genes-16-01369-s001.zip › Supplementary Table S2. DMD deletion.pdf]

Primers

DMD - Exon 5 long range F	**CCCCACTGAGAAACCACACT**	19,234 bp or
DMD - Exon 5 long range R	**CCAAGTCCACAAGAGCCAAT**	2,689 bp

chrX:28,137,793-28,157,026

CCAAGTCCACAAGAGCCAATGCAGCCCACTCTTAAGGTTGTGTATATTGAACTATTAATCATCCAATTATGAAAGATTCAGTA
TATATGTTTCTCAATGGAAGAAAATAAGTATGATTTATGTGTAGAAGTCTTAAAATTTTACATGAATTTTAAAGACAGTATAAAAGTA
CATGCTGCAGGACAGCAAAACTGCTTTTCCTGCATTTTTGATCATTTAAATGCATTATATTTGGAAGTATTACATAAATACTTGAG
GAATTTCTCTTGAATATGGAGAAGTCTCTTTAGCTTGACCGCACAATAGCCGATGTTTTAGAAAAAGTGCTTAACACATTTTATTT
TATTATTTTATTAATTTTTATTGGTGTTCAATTTGCGAACATATAGAATAACACCCAGTGCTCACCCCATCAAGTGCCCCCCTCA
GTGCCTGTCACCCAGTCACCCTCACCCCCCACCTACCTCCCTTTCTACCACCCCTTGTTCGTTTCCCAGAGTTAGGAGTC
TCTCATGTTCTGTCTCCCTTTCTGATATTTCCCACTCATTTTTTCTCCTTTCCCCTTTATTCCCTTTCACTATTTTTTATATTCCCCA
AATGAATGAGGCCATATAATGTTTGTCCTTCTCCGATTGACTTATTTCACTCAGCATAATACCCTCCAGTTCCATCCACATCGA
AGCAAATGGTGGGTATTTGTCGTTTCTAATGGCTGAGTAATATTCCATTGTATACCTAGACCACATCTTCTTTATCCATTCATCTTT
CGATGGACACTGAGGCTCCTTCCATAGTTTGGCTATTGTGGACATTGCTGCTATAAACATCGGGGTGCAG

chrX:28,139,503-28,156,189

16,687 bp deletion (red), exon 5 is in large font and underlined

143 bp of LINE sequence insertion (blue) with sequence similarity (SNPs are underlined)

[AGGTCCCGGCGGCTTAACACATTTTAAAAGCAAATCTTATTTATCTAGAATGCTGGAATGGAATTCTCAGTTGTGAACAATTTG
TCAGAAAGAAATGTAAGAGTATAAAAAAAGAAATAAATAGAAGGTAGAAGCATCTGCATAAGGTCTAAGCATCAGGAGGGATC
TTTATATGATAAATTCCAATGCATGGTATTTTCTTAATTGTTAGTCACTGTTTAAGATTCTGGTTAAGGAAATCATCCAACTTTTAAT
GATGACATTTCATGGTTCCTTTTGAATTCTTGTCATTCTATGTAAAGTCATAAGATTTAATTTACAGCATTTTTTGGTTAAACTCACG
TATTAAGGCTGATGAAGAAAAAAGGATGGCTTACCTTTGTCCCCTACCCCTTCCCTAATACAAAAAGTATTCAGATTGTATTCT
GCATATCAAGGAACTAAAGTGACTTTTGCTCAGTGTAAATCCTTAAACCTGAAATGTCTTAAAGAACCATATCCTTTGAAACCC
ATGTGAGGAACCTCCTGCCCTAACCTAATTTCATTTGGGTGAATGATACAGGATGTGTTTTCATTAGTGCCTAGAGCTGTGAAA
TGTCCTGGGTGGTTGTCTAAAAAGGCAAGTGGCAGATATTGTTCATAGAAGAGATGATGGTTTCCATGTTAAATGAGATAACTC
TACAACTTCCAAGTTAGAACTGTAAATGCTTAAATGATTTACAAACTAAAAGTGGGAAAGTTTATCCATCTCCCTGCTACTGCAA
AAGTTTAAAAAGCGTGAAGAAATTCATAAGAGCTTTATCATAGGAATATCACTCCCTTGCTGTTATTCATGTTTTTTCTTCCTTCT
GCTCTGTCTTAAGAACTTCCATTTTTATACTTGACCAGGCTACTGCGATTATTTTTATGTCAAATGTTCCTCATTCTAAAGTCAGT
GTGTAGCATTTCAAGTTCAATGAACACATTCTGCTATTCCTCTTTCTGTAAAGCTAATTAAAAATAATATAAACTGGCCCAGATCA
GTAATCTCCTTTCACTAAACCATCGTATTTCAGATTTGCTTTAAATCTTATGATTTTCTTCAGTATTCTTAAATCTTAAATATATCAAT
ATAATATATATAAATCCTTTTACTATAACTGCCATGGGATATATAAATTTGGCTTGTAGAAAGTGTTATTGACAATGCTAGATTGCAT
CTATGCCCTGAAGGAACCTGTTGTAAAATAAAACCTGGGATAAAACAAACATTGCTTTCTTGAATAAACAATGAAAAAGAATTG
CTCCTCATAGCCCAGTGGTTAGCACACTCATTTAGGAGGTAGAGCACTAAGAAGTAACAGTGCTCTCTAAAATACTATTTCAG
AGATATATGGTAAATAATATGTGTGTGTGCATATATATATATACATATATATATATTTGCAGATGCTAAGCATCATATTTATTTTTTACTT
TATTTAAAATCAATTAATTAACATATATATTAGTTTCAGAGGTAGAGTTCATTGATTCATCAGTTGCATAGAACACCCAGTGCTCAT
TACTTCGCCTATCCTCCTTAATGCACATCACCCCATTACCCCATACCCCCATACACCTGCCCTCCAGCAACCCTCAGTTTC
TCTCCTAGAGTTAAGAGTCTCTCATGGTTTGCCTCCCTCTCTGATTTTCTTATTTATTTTTCCCTCTTTTCCCTTATGTTCATCTGTT
TTGTTTCTTAAATTCTACATACGAGTGAAATCATGATATTTATCTTTCTCTCAATGACTTATTTCACTTAACATAATACTCTCTAGTTC
CATCCCCATTGTTGCAAATAGTAAAGTTTCATTCTTTTTGATGGCTGAGGAATATTCATTTCTATCTATCTATCTATCTATCTATCAT
CTATCTATCTCACATCTTCTTGATCCATTCATCTGTCAATAGACATCTGGGTTCTTTCCATAGTTTGGCTATTGTGGACATGGCTG
CTATAAACATTGGGGTACATGTGCCCTTTTGAGTCACTTTGTTTGTATCCTTCTGGATAAATACCCAGTAGTGCAATTCCTGGGT
CATAGGGTAGCTCTATTTTTAACTTCTGGAGGACCCTCCATACCGTTTTCCAGAGTGGCTGCATCAGCTTGCATCCCCACCA
ACAATGTAAGAGGGTTCCTTTTTCTCTGCATCCTCATCAACATCTGTGGGTTCTAGACTTGTTAATTTTAGCCATTCTGACAAGT
GTGAGGTGGTATCTCATTGTGGTTTTGATTTGTGAAATACTTATATATTAACCTTAGAATTACTAACTAGATATTTTTGTACAAAATAA
AGTACTTTAGTCAAAGTACTGGTGTGGTCACAAAAAAGAAATTCCCTCTAGTCTGTGTCCTCACATGACAACAATAGACTACTA

TATTTCAAAAGCTAATTCATTAGGTTTCCTACATGCTTTTTTTTTTTGCATTCTGACTCAGGGGATATTGTCCCAAGAGTTCACATT
TAAATATTCTATTAGAGCCATAAGAAGATTCCATTATGGCAAGTCAAATGGTGAGAATTTTCTATCCTTTAAAATTGTTTGAATAAA
GCATCTTGCACAGAGATTGTTGTAAAACTCTGAGCCAGAATGTAATGTTACTTTGTGAGTGAAGTCTAAAGCCTGTTTGTAGGA
ACTCATAAGTCCAATTTTCATGTGTAGGTGAACTTTTGTCTGCAAAAATTCAATGCATGCTATTTAATTTTCGCACACAATATCAA
TTTTCACACTTATTCATTTAGTATCAGATGCTTTCATTCTGCTGAACAGGAAAACATAACTTTGGGTGTCTTATGTGACTTCAACA
TTGAATTACCTGCTATCTAGTGCTTGTTACAATGCACACACACACACACACACACACACACACACGCAGGGTCCATATATTAT
CATATATGTTATATAAATGTTTTTTTGTTTTGCTTACTAGACAGACACACAATTATTCATTTAACACATATCTTAACATATTTCAGTTTT
TGTTTTTCCTTTCCATAGTTAGTTAGAAATTGGACTGAGAATCCTTGAAAGATATAACTCCAAGGCAGATATTAGAAAGATATTTA
AAAGAAGCCATTGTGCATGTGATTATTTAAGAGTAGCAAGTAATAATTTTTTTAATAAGGACCTCATTCATAGCTACATAGGAAAT
ATGGCATTATAAAAACACCAAGCATATGAGAATTTAGAGTCAAAATGGGATAGACTGTCGATATTAGCATTCAATATTAACAAGA
CCATGAACAGTACGTTTTAGGCAACAAAATACCCCTCCCTCTTCTAGAGCCTTAGTGACTATGAATTTCTTATAGTCCATGGTA
ATTTGAAGAAGAGTTGTACAGCAAGAGACAAAACACAACCCAAACAAATAATATTCCTTGTCTACGTGGTGGTGTGCAATATTT
ATTAATTTAGAGACAGCCTATTAAAGCACTTCATGAAGAATTCTCTAGATTTAATTAAGGAATTATCTCTCCTATCCAGACTTGGT
TTTAAATCTGATCTTGTGAAAGCTTCATGTACCTTTGACATTCTGTTTTCCTCTAGGTAAAATGTATATAAAATAGTAACTATCTCAT
ACTTTTACTGTGATGATGATATGCGTACAGTGAAAGGTGTATTCAGGGAGAACCTTATAGGCTTAATGTTTTTTAAAGATAGAGA
CAAAATAGGTATATATCTTAAGAGATTATAAGAAGAAAAACAATAATGAAAAGTAAATTCAAAATAAAAGACTGAAAAATATAGGT
GGGATTGCATTCAAAAGCTAGCTCTTTGAATAGATCAACAAAAGAGATAAATCTCTTCTAATCCTAACAAAAGAAAATAAGAAG
AGAATGAAAAGTAGAAGAGATTAGTAATGAGAAAGGATGTGTAACCACAGATTCAGAAGAAACTGAGAGAATACTACAAGCAA
TTCTGTGAAGCTAATTTTGAAAAATCTGGGACATGGATGATTTCCTAGAACAATGCAAATTACTAAAATTGACATAAGAGTAGCA
GAGAATTAAACTAGACTAATTACCTCTGAAAACACTGGTAAGTATTAGAGATCTACCATGGAAATATATGCCAGGATTCTTCACA
TATGAATTTAACCTAATCCTGAAAGAATAAGTAATTCCTATATTTTAAGGTATTCCAAAGCATATAAGAAGATAAAACCCTGCAAT
TCCTTTTGTGATGTCAGAATAACCATAGTACTAACAGTCAATAAACATAAAATAAAATAATAAATGTATAAGACAATCTCACTCAT
GAATATGAATATAAAATTAAATTGTTGAACTATATAAAATTGATTAAACCATATCTTAAATGTGGTAAGTACTCAATAAATGTTATCTA
TTAGTAGCAAAGAGCTCTTAAAATTAAGAAGGAAAAGAAAACTAACCTATAGAAAATGAATAAGCCATATTAACAGTTTACAGAT
GAAATTGAAATAATCAGTCATACAAAAAGATATTCCAAATGTACTGGGAATCATGGAAATAAATTAACAAGGCATTATCTTTTCG
CTCATCAGACAGGCAAAATGAAAAGGCAAAAACATCTATTGCCGGTAAGAGAGGAGCCTATTCATATACATCACTGGTTAAA
AGACATGCTAAAACCTTTTTATAAAGAAAAACCTACCAAAATTAAAAAAATATGTATACATTTGGTCTAGTAAATACATTCCTGGG
AATAGCTATTATAACAATAAAAACATCAGTTATCTGGGGATGTATATACAAGTTGTCCATTGCAATATTATATAAAGTAGCAAATTA
AAACAAAACAAATATTACAAGTATAGAAACAAAATGAATGTCCACTAACATGGGACTGGCTAAATACTCTAGGATACAACCAA
ACTATTAACTATTATGCAGCCATTAAAATGAATGAATAAAATGGATTATTGTTTGGTAAAAGCAATAATGAATAGAATCTGAATAAT
ATGCCATTTTGGTAGAATATAAAACCCTATAGATTTGTGCCACATGCAGTGATCATATGAATAGGAGAAGAATATGAAACTTTTTT
TTTAGGCCAGATTAGGAAAGAAGTCAGGGAAAGAAATAGAGGCAAACAAAATGGAAAAGAGCTTGCTTCAAGAAAGTTGGG
AGAGATGCATGTATGAGATCTCATGCATTTGTGTAAAATATATTCATGTGACTATATATAAAGCAATAAAATATTTCTAAAATGAACA
AAAGTAGACTATATATATATACACACACATAAATAAAAAACAGCTCAAATATTTGCCTATAGTTTGATACTCAAAAAATGGTTATGG
TTGTGTTTCTTACAACCCAACTACAGGATGTCAAAATACCCCCTTATGGCAGCTTGACTTGATCAAGTTTGGAACTTTAGAAAG
GAATGATGACTGGTACTAATTGCAGTGTGCTAAAAGCCACCTCCTAAGGCATACAAAATAGAGAATATATGAGTGAAACACCA
TTTATAAACCATGGTACTCACTCATTATGCTCCAGGGCAAGGACCACCAGTGAAACAGAGGTGGTCCGTGCCATAGTAGGA
CCTGATGATATAGTGATTGATACTGGCAATAAAAAAAGTAAACTAACAAAATATAATTTTAAATCAACAAAGTTACATAAATTGGAT
TGCATCATTGTAACTTTCTATAGACTGCATGAAACTAAGATTTAATACTCTGCTCTTCTGTTTACTGAAAGCTGACAACGGAAAT
GGCTCTATGGTTATCACCATGTTACTTGTCAAGTTGTAATTAAAGATGTAGTATGTTATTATAAAATGCACATTTGTTAGAGCTCAG
AATTGGGAAAAAGGTGGTTGTCCCTGAGAAAAATTTAAGCTTCCTTGTATTAGTTTAAGACATCTATAATTGATCTATGCTATCTTT
TTTTAAGATTTATTTATTTATTCATGATAGACACACACACACACAGAGAGAGAGAGAGAGAGAGAGAGAGAGAGAGAGAGAG
GCGGAGACACAAGCAGAGGGAGAAGCAGGCCCCATGCCGGGAGCCCAACACGGGACTCGATCCCGGGACTCCAGG
ATTGCACCCTGGGCCAAAGGCAGGTGCCAAACTGCTGAGCCACCCAGGGATCCCCCCCATGCTATCTTTTTTAGGGCCT
GTAACAGTTAGCCAACATCTGCACAACTTTTCTGCTACTATTGCCTCTCTACTTCATTAACTCCTCCCTATAAGTCAGTTCCTTA
TATGAATTGAGATTCAAGTATTCTTCATAATCCTGTCCCAGATTGTTTAACAATTTCACCCCATGCCACATTGTCCCTGAGCCC
AATACTTCTAATTCTCAATCAGGCAATATCCTTTACTCTATCCCCATATCTTTAGGCATGTGGGATCTTCATGGCAGGCCTCCA
CCTGCCACATTCTTTCTTCCAGCCTTCATGTTCCTTCCTTCACAGATTCAATCAATATTGAATATTTTGTCAAATGCTTTCTCTGC
ATCTATTGAGAGGACAATATGGTTCTTGTTTTTTCTCTTGTTGATGTGATCTATCACACTGATTGTTTTACAAGTGTTGAACCACCC

TTGCATCCTGGGGATAAATCCTACTTGGTCATGGTAAATAATCTTCATTTACTGCTTGATCCTATTGGCTAGTATCTTGTTGAGAA
TTTTTGCATGTGTGTTCATCAGGGATATTGATCTATAAATTCTCCTTTTTGGTGGGGTCTTTGTCTGATTTTGGAATCAAGGTAATG
CTGGCCTCATAAAGCAAGTTTGGAAGTATTCCATCCCTTTCTATCCTTCAAAACAGCGTTAGTAGAATAGGTATTGTTTCTTCTT
CAAACGTTTGATAGAATTCCCCTGGGAAGCCATCTGGCCCTGGACTTTTGTGTCTTGGGAGGTTTTTGATGACCACTTCAATT
TCTTTGCTGGTTATTGACCTTTTCAGGTTTCTGTTTCTTCCTGTTACAGTTTTGGTAATTTGTGGCTTTCCTGAAATGCATCCATTT
CTTCTACATTGCCTAATTTGTTGGCATATCGCTGCTCCTAATATGTTTTAAAAATCGTTTGTATTTCCTTCGTATTGGTTGTGACCT
TTCCTCTTTCATTTGTGAATTTATTAGAGTCTTTTCTCTTTTGTTTTTAATAAGGTTGGCTAATGGTTTACCTATCTTATTCATTCTTTT
GAAGAACCAACTCCTGGTTTTATTGATCTGTTCTACAGTACTTCTAGTCTCTATTTCATTGAGTTCTGTTCGAATCTTTATTACCTC
TCTTCTTCTGCTTGGTGTAGGTTTTACTTGCTGTTGTTTCTCCAATTCATTTAGATGTGAGGTTAGCTTGTATATTTGAGTTTTTTCC
AATTTTTCAAGAGGCTTGTATTGCAATATATTTCCCTCTTAAGACGGCTTTTGCTGAATCCCAAAGATTTTTAACAGTTGCATCTT
CATTTTCATTAGTTTCCATGAATCTTTTTAGTTCTTCCTTAATTTCCTGGTTGACCCATTCATCTTTTAGTAGGATGCTCTTTAACCT
TCATTTGTTTGAGTTTCTTCCAAATTTGTTCTTGTGATTGAATTCTAGTTTCAAAGTATTGTGGTCTGAGAATATGCAGGGGACGAT
CTTAATCTTTTGGTATTGTTTGAGACCTGATTTGTGACCCAGTGTGTGGTCTATTCTGGAGAAAGTTCCATGTTCACTTGAGAAG
AATGTGTATTCAGTTGCATTCAGATGGAAAGTTCTGTGTATATCTGTGAAATCTATTTGGTCCAGTGTATCATTTAAGGGCCTTGT
TTATTTGGTGATGTTCTGCTTAGAAGTACCTGTCATTTGCTGAAAGTGCCATGTTGAACTCTCCTACTATTAGTGTATTATTTATCT
ATCTCTACTTTATTAATTGATTGATATACTTGGCAGCTCCCACATTAGGGGCATAAATATTCATGATTGTTAGGTCTTCTTGCTGG
ATAGACCCTTCAAGTGTGATATGGTGTTTCTCTTCATCCCTTACTACAGTCTTTGTGATAAACTTTAACTTATATGACATGAGGATT
GCTACTCCAGCTTTTTTTGAGGACCATTTGAATGGTAAATGGTTCTCCACCCCTTCATTTTCAGGCTGGAGGTGTCCTTAGGT
CTCAAATGTCTCTTGTAGACAGCAAATAGATGGGTCTTGCTTTTTTATACAGTCTGAAACCCTGTGTCTTTTGATGGGGTCATTT
GACCCATTCACATTCAGAGTAACTATTGAAAGATATGAATTTAGTGTCATCGAATTACCTATTCAGTTCCTGTTTTTGTGGATTGT
GTCTTTGGGCTTCCTTTCTTTTACAGGGTTCCCTTTAATATTTCTTGCAGAGCCTGTTTGGTGGTCACATATTCTTTCAGTTTCTG
CCTATCTTGGAAGCTCTTTATCTCTCCTTCTATTCTGAATGAGAGCCTTGCTGGATAAAGTATTCTTGGCTGCATGTTCTTCTCAT
TTAGTACCCTGAATATATCCTGCCAGCCCTTTCTGGCCTGCCAGGTCGCTGTGGAGAGGTCTGTGTTAATCTGGTATTTCTCC
CCATTAAAGTTAAGAATCTCTTGTCTTGCACTGCTTCAAGAATTTTCTCTTTATCTTTGAAATTTGCAAGTTTCACTATTAAATGTC
GAGGTATTGAATGGTTTTTATTGATTTTTTAGGGGTCCTCTCAGTCTCTTGGATCTAAATGTCTGTTTCCTTCCCCAGATTTGAGA
AGTTCTCAGCTATGATTTGTTCAAATATCCTTTGTAGTCCTCTCTCTCTCTCAGTCTCTTCTGGAATCCCAATTAGATGTATATTCT
TCCTTCTCAAGCTATCATTTATTTCCTTAAGCCTTTCTCAAGGGGTTTTAATTGTTTTTCTCTTTTTCCTCAGCTTCCTTCCTTACC
ATCAGTTTGTTGTCTATGTCACTCACTCTCTCTTCCACCTCATTAACCCTAGCAGTTAAAACATCCAGTTTGGATTGCATCTCAT
TTAATCTATTTTTAATTTTGGCCTGATGAGATCTCAATTCTGCTGTAACAAAGTCTCTAGAGTCCTTTATGTTTTTTTCCAGAGCCA
CCAATAACTTTATAATTGTACTTCTGAATTGAATTTCTGACATCATATTAAAATCCAAATTCTGTAACTCTGTGGCAGAGAGTACTG
TTTCTGGTTCTTTCTTTTCTGGTGAATTCTTCCTTCTAGTCATTTTGTCCAGTGCAGAGAGGCTGTTTAGGCGGGCTGAGTCAA
AAATATCAACCACGACCCAAGTAAAATATACCCTAGATGATTCTGAAGAGGTTAGAGACCAGAAAATAAAAGAAAAAGAAGA
ACAGAACAAAATAAAAGGACCACTAAAGTGAAAAACAGATTTTAAAACAAAGTAGTAAAAATAAAAAGCCAAAAACAAAGAA
GAAGAAAAAAGAAAAAGAAAAAAAAAAGTAAAGAAAAGAGGAAAAAGAGAAATAAAAGGGGGGGGGATGGTGGTGGTGAG
GAAATGGTAGTGGAGAGAGAATATAGTCTACCTGAGGGGTCCTAGAGGGTGATCTTCTTGGTTCTGAGTGTATTTTGTTCTGTA
TGTTAGCAGATGCTTAACCCCAAATTTATATAAACCAGAAATACTTATATAAAGCCCCAACATTGACCACCAAAACATAAACAA
GATAAAAGAGGAGGAAAGAATGGGAGGAAGAGAGAATTTAATCTCACAGAATGCCAACAGGGTGTTCCACTTGGTTTCAGG
TGTATGCTGGTCGTGTTTTAGAAGGTACTAACTTCCACCACTGTAAAACAAAATGAGGCAGAAAAAAACAAAAAACAAAAGC
CCATATGCCGTATATCTACCAAAATTAAATAGAATACATTGAAGGGAATCCAGAAGTGAAAAATATATCTAAGACATGTCATTGT
AGAAATATGTAAGTCAAAAAGGAAAAAACTTGAAAATGAAGAGCTGGTAATATATTGTAGTTAAGGTGGGAAAAGAGAAAAAAT
ATTGGAAATTTTAAATCTGATATAAAAATGAGTCATAATGGAAAAAGAAAAAAAAGTAAAAAAGGACTCTCTAGTTCTATATACTAT
TTTCCCTCAGTCCTGTAACTTTCCAGCACTTCTTGGTCAAGAACTTGTTCTTACCCTGTTCTTCCAGCTGGTCTTCTGGGGGA
GGGTCGGCTGTGCGGGTTCTCAGGGCCTGGGCCGAGATGCCCCGCCCCCTGCTGGGTGCCGGGCTCAGTGGGAGCT
GTTTACCCCATGAGGCCTTTATTCCCTGGCGGCCCTGCCTCAGAGTGATACAAGGCACAGAGTGATACAAGGAGGAAAAG
CAACGCAGGCCACGGCTAGATTTCCAGATCTGGAGTCGAGCTCCCGGTGAGTAACTAAAGCAGTCTCCCGGTCCTCACT
GGCCTAGATGCTCCCAGGGCCAGCATGGGTGCACTGATCTGCACAGCTTGTGGGGTGCCAGGAAAGTTCTTGCTGTCCT
GCACCCTCCTTGTTTCCTCCTGTCCTGGGGGGAATGCAGGATCGACAGCTTTGTCCACTTGGGCACCCTGGGATCTGGG
GCCAGTGCTGCTGGACCTGTGCCTACAGGGACCATCTCTCCCAAAGGGAACAGGGTACAGCCACCTCCCTCCGGAGCC
AGCCTGCCTAACCAACTGGCTTCTCCCAAATGCCCCGGAGGGTTGCAGTTCTCCAGCCCTTTACCTTTAAGGACCCCG

GTTTGCGGTGAGCTTTCCCCTGCAAGCCCCTCCTCTTTTAGTGACTCTGGGAATCTTGAGGCTTCATTGTCCCTTCTGTGATT
CTGCCCAATTTCCCTGCTAAGCACTTTTCTGTCAGGGAAAACTCTGGTGCGGACTTTTAAAGTTCCCACTTCTCCAGGGCTC
AGCTTTCCTGCCCTGGAGGCTCTCCCCGCTCCGCTTTAACCTGGCTACTGGTGGCTCCCCTCCCCCATTTTATTCTTTTTTT
ACCCCCCACCTTCCCACCTTGTTAGAGGCAAAAACTTTTCTCTCTGTAGCATTCCAGCTGTCCTCTCTTAAAATCTCAGGTC
GAATTCATAGGTGTTCAGGATGTTTTGAAAATTATTTAGGTGTTTGTGGGGCCAGGTGAGTTGAGGACCCCTACTCTTCCACC
ATCGTCAACATGTAATATCTTATAATGGCAGTATCACTTTGACTTTGCTCTATCCAAGAAAGTCAAAGTCTTCATGTTAACTAGTTT
TCAGGACTTCCATTTATCCACTCCTCAGCTGAACCTTAGTGAAATGTCCATTTACATACTACTTCAGGCTGACTCCGCTGTGA
AGTCCATGTGGTACCAATCCTCACATCTAATCTATTGCATAGCTCAGTAATATCCTCTACTATAAAATATATTTGGAATTAAACTAT
GGACTTATACTTTGATCGTCTATCCTTATTCAAGTGATTCTACATTCTTTTCTGTATTCTTTATTTGTTTTAAGCCATTACACCAATAA
TGTTTATATAGTTGGGATACAAAAATAAATTTGAATATTTGATTATCCATTGGACCTGTAAGTAACTTTATAGAAGGCATAATTGGG
AGTGATTTAACAATTATACCAAATGGTAGCCTTTTTATTGTTTTCACCTTGATTTCCCAGACAGCTTCATATTTTCAAGTTGTGATT
AATAACATCTAGAAAATTTAGATGAATGATAAGGAATATAATTTATCATTGAGTGATAGAATGGTGGACCCTTCTTACTGAAGGAA
GAAACTAATGGAAAAGACAGGTTGGAGTGACATGTAGACCAGTAAGGGTTTGGAGCCTTAGAAAATGAAAGGTTTAATTAAAA
TGACTCTTGGCTATTTAGAGAACATATCCATGGGAAGATGAGGATTAGGCAAGTCTCTCAGGTTGCTGCCCTTAGGATAAGAA
AAAATGGTAGGATCCTGGAGGCAGCCAAAAGAAATTCAACCAAAAGGATCAGTGAGCCAAGAATGGTAAATTGGGAAAGA
AATATGAGTTACTTCTTAACCCTCACTCCTGCTCTGACAGTTTCACATCCACTCTACAGACCTTAATCTGAAGTTGCCTATTTAC
TGTGATATTTTCTCCTTTTAAAGTATCCCTGGATGTCCATTTCTTAGTTAATCTTTTTATTTTGAGATAATTGCAAATTCACATATAGT
TGTAAGAAATAATACAGAGAGATCCTGTGTTCCTGTAGCCAAGCTTTACCCCAATGGTGGTATACTGTAAAATTGTACAATGATA
TCACTACACATATCAACATTGATACAAGCCACCAATAGTATTTATTATTTATTTATTTATTTATTTATTTATTTATTTATTTATTTATT
TTAGTATTCATATTTCTTGAGTTTTACTTGCACTCAAGTATGCATGGACAGGTGCTTTAAACTCTGTACATTTTCACATCTGTGCAT
GTGTGTCTCTACCATCACAGACAAAATGCAGAACAGATTCATCACCACAAGGATCCACCACTATGCCATTTTAGAACCACAT
CCACCTACCCCACTCCCTCACCCTGATCTCTGGTATCAATTGCTTTAACTTTGTGTATTCAAAACCATTATAGTTAATCATTGTA
TCTGAAACCCTTTGAGATTTGCCTTGTTTTTTCAACTCAGCATAATTCCTATGAGATTTACCCAAGTTGCTGCATTTATCAACAGT
TTATTTTCATTGCAGAGTGGGATTCCATGGTACGGATGTACTACAGCTTGTTTAACCCTTCACCCCTGAAGAACAACTGGGCT
GTTCTCCCAGATGGCTGTGTTTGACCATTACAAATAAAGCTGATATGAACATGTACACATAGGGTTTTGTAAAAATATAAGTTTT
CATTGCTTTGGGATAAATGCCCAAGAGCCCAGTTGCTGGATTGTATGCTAAGCCCATGTTGAGTTTTTTTTTTAAAGACTTATTT
ATTTATTTATTCATGAGAGACACACACAGAGAGAGGCAGAGACATAGGCAGAGGGAGAAGCAGGCTCCTCGAGGGGAGC
CCAATGTAGGACTTGATCCCGAAACTCTGAGACCACACCCTGAGCCAAAGGCAGATGCCCAGTCGCTGAGCCACCCAC
GCATCCCCCATGCTAAGTTTTGCAAGAAAGGACCAAAATGTTTTCCAGGCACTCTGGTCTGTACTATTTACATTTTCCCCAGC
AATGCGTGAGTGATCCAGTTTGTCCATATCCTCACTGGCATTTGATGTTGTCACTATTTAAAAATTTTAGCCATTATGATAAGTGC
ATAGTGCTAGCTCATTGTGGTTATAATTTGCTTTTTCCTAATGGCTAATAATGTTGAACATCTCTTCATTCACCTATTTTATTTGCCT
GATGCTTATGTTTATTCTGACTCCACCTAAAAATATTAGGAACATAAAGGACAGGAGATAGAATTATCCTTAAAAAAAAATAGCC
TGCCAGATTTTGATCCCCAAACTTAAAGCGAAGAGGGCTCCATTATTTATTAGAGTTCTAATATCTGTTTCTAAAGCAAGGCCA
TTTTAGTCAAATGCAGTCACCCCTAATTGTAATAGCACTCTTTTGAGCAAGATTAAAGGACTGAGGAAATTTCTTTTTATTGAGTT
TCAATTTTGTATTAGGAAGCACATCTTAATTTTAGGCATCCTTTATGAGTTATCATTGTTATACATTTTATTTGAAGATATTCTGATTG
GCCTCTTTAATCTGCAGTCATCTGTTTATATACTAAAAGTACATTCATTTCCTATCTCTTTGCAAATGTGTTCAGGGGCACTTGTTT
GTTAAAGAGAGTGCATTTACAAATGCATTTTGTTTCATTTTTAGATTATTTTTTAAAAGCTGTAATACTGTTTAGGCTCTGAAAATAG
CTAAATGCAATTTTGTTTTCTTCCCAAATGGACATTTTGTCTCTTTCCTTTAAATGTTTTCCCTTTATCTTTAACAG**GTTGATTTA**
GTGAACATTGGAAGTACTGACATAGTAGATGGAAATCACAAACTGACTCTTGGTTTGATTTGG
AATATAATCCTCCACTGGCAGGTAAGAATCCTGATGAATGTTTTCTTGTTGTGAAAAATGTTTCTGATTCTTCCTTTCA
TTTTTATTCTTGACCTGCAAAACAGACATATATACCTCTGGCAATACAAAGCCCATCTCCATAGCACTGCCTTTTAAATGGGCA
CGACTGACGTCATTTGAGATGGGATGGATCAGCAGATTATAAAACAGATTCTAGAACATTTGCAGGAATATTTTTCTAAATTTTA
CTTCAATGTGTGGAGATGAGACAAAAAAGACCAATCTCATCAATAGGTTAATAAGACTATTTTTTCTCAAATATATGTCTTTTATAA
CCCACCTATGCATGAATCTACAGTTTTGCAATAAACTGTTAATCACTACCAAATGAATGAGACTGTGAATTTTTTGTATTCTCAAT
ATAAATTAATGCCAGTAAAAGATTAGTGGGGGATCCCTGGGTGGCGCAGCGGTTTGGTGCCTGCCTTTGGCCCAGGGCAC
GATCCTGGAGACCCGGGATCGAATCCCACGTCGGGCTCCCGGTGCATGGAGCCTGCTTCTCCCTCTGCCTGTGTGTCTC
TGCCTCTCTCTCTCTCTCTCTCTCTCTGTGTGACTATCATAAATTAAAAAAAAAAGATTAGTGGGATATGATTATCTATATATATATGTT
ATACATACAAATTAACTTCATAATCTGATTTGGGAATTGCATTTTTTTTCTGAACACTCAGCAAAAGGTTTTCTGAATGTGTATATAT

ATGTTGGACCTCTGCAAAACAGATCAAAGGTCATTTGGTTTTAGTTAAGAGTATGCATTTTTTCAGTCAGACCAACATGAACTTA
AATTTCAACTCCCCCATTTACCATCTTATACTGACATTGGGTATAATGAAATCCCTTTTGGGGCTATACTCTCTTTTTTTTTTTTTTT
TTTTTTTTTTTTTATTGGTGTTCAATTTACTAACATACAGAATAACGCCCAGTGCCCGTCACCCATTCACTCCCACCCCCCGC
CCTCCTCCCCTTCTACCACCCCTAGTTCGTTTCCCAGAGTTAGCAGTCTTTACGTTCTGTCTCCCTTTCTGATATTTCCCACA
CATTTCTTCTCCCTTCCCTTGTTTTCCCTTTCACTATTATTTATATTCCCCAAATGAATGAGAACATATAATGTTTGTCCTTCTCCG
ACTGACTTACTTCACTCAGCATAATACCCTCCAGTTCCATCCACGTTGAAGCAAATGGTGGGTATTTGTCATTTCTAATAGTTG
AGTAATATTCCATTGTATACATAAACCACATCTTCTTTATCCATTCATCTTTCGTTGGACACCGAGGCTCCTTCCACAGTTTGGC
TATCGTGGCCATTGCTGCTAGAAACATCGGGGTGCAG]

GTGTCCC**A**GCGTTTCA**C**TGCAT**C**TGTATCTTTGGGGTAAATCCCCA**G**CAGTGCAATT**T**CTGGGTCGTAGGGCAG**A**T**C**TATTT
TTAACT**C**TTT**A**AGG**G**ACCTCCACACAGTTTTCCAGAGTGGCTGCACCAGTTCACATTCCCA

[CCAACAGTGTAGGAGGGTTCCCTTTTCTCCGCATCCTCTCCAACATTTGTTGTTTCCTGCCTTGTTAATTTTCCCCATTCTCA
CTGGTGTGAGGTGGTATCTCATTGTAGTTTTGATTTGTATTTCCCTGATGGCAAGTGATGCAGAGCATTTTCTCATATGCATGTTG
GCCATGTCTATGTCTTCCTCTGTGAGATTTCTGTTCATGTCTTTTGGGGCTATACTCTCTTGCTGTCAAAACAGAAAAAAAAATA
ATTAACATGTATATTTCTTGTCAGGATTGAATGAGAGAATATCTGTAAAGCACTCAGTGTAGTACATCATTGTCACCATAGGTGTA
TCATATGAATAATTTTGATTTCTTGGAGTTTTCTACTCATATTTTAAGAATCTGGAGGAAAATTGGGATGTCTGGTCTTTTACTAGC
AGTGATTAATTTCTTTATAAAACAGATTTTTATATGTAATTTATGTATCATAAATTCACCCATTCTAAGTATAAAATTCAAAATGTATCA
ACTGGTTTAGTTCTCTTGCCCAGTGCCTCTCAAATTATTTTGAGTAAGGAACAGTTTCCCCCCCCCAATCTGTCAAAGCTTTT
GGGATTTTTACAGGGTTGTATAATCATAAGAACAATCTAATTTTAGCATACTTCCACCAACCTAAAAACAAATCTTGTCTCTTTAT
AGTCACTCCCCACTCCAAACCCCAGCACTGGGCAACCATTTATCTGTTCCTATGTCTGTCTTTTTGGATATTTCATGTCAATGA
AATGATATAGTAAGTGTTCTTTTATACCTAGTTTCTTTCACTGAACATAGTATTTGAGGTTTATCCTCATTGTAGCAAGTATCTCTAC
TTCATTTAGTAGGGTTGACTTAACTTGAATTGTACTTTGCTCCTGCTCAGAGTCAGTGGGTAGACCAGAGGTTACCTATTCAAC
TCTGCAGTTGAAATGAGCCCCATATTGACCCAATTGACTCCCCTACCTTTTTATTCCAAATACTCGGAACAGGCTCAATCCTA
ATTCACAGCCACATGGGAAGAGATAGAGGACAGCATAGTGGATGACAACAGAAAATAGACCATAAGAACCACAAATGAACC
TGAGATTCCCAATTCTGGGCTTTTTCCATATAGCAATGGTATCAAGTGGAACCAGCAAG]

chrX:28,137,793-28,139,501

CAACACAGGACCCTTCACCAAACTGGTTTACTCCTGTTGCTTTGTCTTGAGAAAAGAACAGTTTTTCCCTCTTTTTCATTGTAA
ATTATAATAAAACTGAAGCATTACAAAAATGAAATAAAAAGACACACAAAACCCAAGTCATTTTATAATTAAACTCAACAGACAT
ATGTAGGACCTTGTAAAATTGTTGCAGAAGTTTCTAATGGACTATCAATTGATTTGTCTTATGGCGTGGTTACAAATGGTTCATGG
AGGGACCCCATCTTGCAGAAGACACTAAAGTTGTGTAGATGGAAAAGTTAGGCATGCACAGAGACCAGAAGGCAATTTTAA
TTGTATTTTTCATCCAGACTATATTTCATAGACAAGCACTTTCTTGTTTTCTCAAAACCCTTTCCAACTCCATCTCAACTCACTGC
AATATAGTTTCAAAACTACCCTGATACTTTTTTCATTTGTTTGTGAGCTTGCTCATTTTTAATAATTAACATGTGGAGGGGCTTTCA
CATTTGGGAATATCAGAAGGAATGCTTTTGTAGTCTTTCCTTGTCTGAGACCTCTCTTACAGTCGTCTGTTGTTTTACTCCTGTGT
CCTTGAATCACCATATCCTTGTGATTGCAAGGATGTAGACTCTTCTCAGCCTCTGCCTCTTTATCTTATCTCTCTTCTATCACAC
TGCTTGGTCCTTTTAAAACTACTAAACACACTTAAAAAATTCTTCCTGCTCAGTACTCTTCATCATAACCCATTGAATCCTAAAA
GGTATTTTCACCAAGAGTACATATTTGCCTTCCTATCAAGTAGTCAAAGAAGCCCTTGAATCTCAGCAGAGATTAAAGGACAC
TCTGGCCAACCTTAGGTATTTTCCAGATCTATCAGATTGAGAATGACATTAAGAGATGGAAAGATGTGAATCTTTTGTTCCAGTG
CCACATACTATGTTATTAATAGTGCCATGGAGGACTTCTCTTTGAACTTGTAAAAGGTCAGGAATGCCTACCCTCTCTTTCCTAT
CACTGTTTTTCCAGTTTCCATGTACATAAAATTGTTAGCCTGAAAAGTAATGCCTATATTGAGGAATAAGCAGCTTGAACAAAGT
TGTGAAGTTGACGGTTTGTGTTTGGCATCTTTTTCTTCCTTCTTTTTTAAGTGCAAACCATCCTTTTTAGGGTGGTCAGTTTGGTT
CTGACAACCAGCTGGAGATACAAACATACCCTGTTAACAATGGTGTGCTCTCATAGGTCAGATCTGACCTTTGCTGGCTAAG
TCAAAGGAAATATTCAAATCCAGGGCTACTTTACACTGCAATATTTATTTACTTATTATTTGCCCATCATGCAGGAGAAAAATGG
GTTTTACATTGACGGAAGACCTTGCTGTAAACTGCCTACCTAGTATCCTGTGTGCATTAGTCTTCTTCACACACAGCCTTTCTT
TGTAGAAGAATTATCCCTACCCGTCTTTACTCCTACCCCTTCACCATGACTCCTTTCCATCACGGGATGGACTGTGGGAAGT
AGATGTTGAATTGAGCAGGCAGGACATTAATTGAAGCATACCTTTGGGACCAGCACTTGAGGAGGGAAGAAGTTAGCTGAA
GGGAGAAGCACTGAAGCTATGATGTACACCCAGCCACAGTGGTTTTCAACCCCACTGGGAACTGGGAGCTAGAATAGCAA
CCTTTT**AGTGTGGTTTCTCAGTGGGG**
